# Vacuolated Leukocytes in the Peripheral Blood Smear of a Child with Chanarin-Dorfman Syndrome

**DOI:** 10.4274/tjh.galenos.2020.2020.0242

**Published:** 2020-11-19

**Authors:** Arzu Akyay, Filiz Demir Şahin, Aşkın Şen

**Affiliations:** 1 İnönü University Faculty of Medicine, Department of Pediatric Hematology and Oncology, Malatya, Turkey; 2 İnönü University Faculty of Medicine, Department of Pediatrics, Malatya, Turkey; 3 Fırat University Faculty of Medicine, Department of Medical Genetics, Elazığ, Turkey

**Keywords:** Chanarin-Dorfman Syndrome, Vacuolated leukocytes

## To the Editor,

Chanarin-Dorfman syndrome (CDS) is a rare autosomal recessive inherited neutral lipid metabolism disorder (NLSD) characterized by lipid vacuoles in leukocytes (Jordans’ anomaly) on a peripheral blood smear and ichthyosiform erythroderma with involvement of multiple tissues in the body including the liver, skin, muscle, eyes, ears, and central nervous system. More than 100 cases have been documented worldwide, mostly from the Mediterranean region and the Middle East [1,2,3,4].

**Figure 1:**
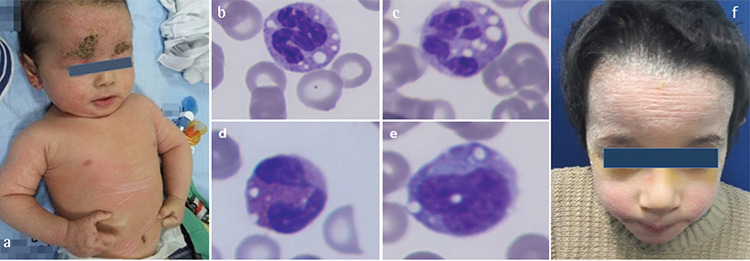
Non-bullous ichthyosiform erythroderma on the scalp, face, trunk and flexures (a), lipid vacuoles in leukocytes (b, c, d, e), persistent ichthyosis (f).

A 4-month-old boy with congenital ichthyosis was referred to the pediatric hematology department because of leukocytosis and thrombocytosis. It was learned that the baby was born at full term as a collodion baby by cesarean section as the first surviving child of the family. In the family history, one brother had died of respiratory failure when he was 10 days old. There was consanguinity between the parents and there was no similar patient in the family. Physical examination was normal except non-bullous ichthyosiform erythroderma on his scalp, face, trunk, and flexures (Figure 1a). A blood count revealed leukocytosis (18.5x10^3^/µL) and thrombocytosis (674x10^3^/µL). Biochemical examination results were normal, except for mild aspartate aminotransferase (AST) elevation (75 U/L). The peripheral blood smear revealed vacuoles in neutrophils and monocytes consistent with Jordans’ anomaly (Figures 1c-1f). Serum lipid analysis showed elevated serum triglyceride of 257 mg/dL (normal range: 0-149) and very-low-density lipoprotein cholesterol of 51 mg/dL (normal: 10-40). Urine culture revealed *E. coli* (>100,000 CFU/mL), and the patient’s leukocytosis and thrombocytosis were thought to be due to a urinary tract infection. Due to the presence of ichthyosis and vacuoles in leukocytes, CDS was suspected and genetic analysis was performed. *ABHD5* gene analysis revealed homozygous c.594_595insC (p.Arg199Glnfs^*^11) mutation on exon 4, consistent with CDS. This mutation was previously identified (HGMD number: CI013354) in a Turkish family and in a child of Kurdish origin [5,6].The *ABHD5* gene encodes a protein that is a member of the esterase/lipase/thioesterase subfamily. To date, 37 different types of *ABHD5* mutations have been reported in CDS patients [7].

In CDS, lipid droplets accumulate in the cytoplasm of various tissues as a result of abnormal catabolism of triacylglycerols, without affecting lysosomes. Non-bullous ichthyosiform erythroderma or fine scaling on erythematous skin since birth is a characteristic finding of CDS [8]. In addition to skin manifestations, steatohepatitis, sensorineural hearing loss, sub-capsular cataracts, nystagmus, strabismus, myopathy, and mental retardation can also be seen. NLSD skeletal myopathy and cardiomyopathy are two clinical variants of NLSDs in which ichthyosis is not observed while vacuoles are detected in leukocytes [9].

There is no specific treatment for CDS. However, a diet low in fatty acids with medium-chain triglycerides (MCTs) was reported to decrease hepatomegaly and normalize hepatic enzymes, especially when initiated early upon diagnosis with the combination of vitamin E and ursodeoxycholic acid [4]. Emollient cream, artificial tear drops, and a diet containing MCTs were began for our patient, and he has been followed for 5 years since the initial diagnosis. His thrombocytosis and leukocytosis improved and complete blood count values remained in the normal ranges until 5 years of age. At the age of 5, the patient had persistent ichthyosis (Figure 1b) and was diagnosed with mild mental retardation and multi-organ involvement. The liver was palpable 3 cm below the costal margin with moderate increase in transaminases (AST 118 IU/L (normal: 0-40 U/L); ALT 134 IU/L (normal: 0-55 U/L)) and there was no splenomegaly. He developed grade II hepatosteatosis at the age of 2.5, punctate keratopathy at the age of 3, and mild hearing loss at the age of 5. Myopathy has been reported in a patient with the same mutation as our patient [6]. However, our patient did not have myopathy.

Besides NLSDs, leukocyte vacuolization can be seen in acquired conditions (infections, alcoholism, toxin exposure, diabetic ketoacidosis) and in inherited conditions (carnitine deficiency, multiple sulfatase deficiency, Refsum disease, Wolman disease). In acquired conditions, leukocyte vacuolization is temporary and there is no accompanying ichthyosis. However, in the inherited conditions mentioned above, there is persistent leukocyte vacuolization accompanying ichthyosis, except in cases of carnitine deficiency. We excluded these disorders in our patient with the presence of *ABHD5* gene mutation [6].

In conclusion, CDS should be kept in mind in cases of patients presenting with the combination of marked ichthyosis and persistent leukocyte vacuolization in blood smears.
